# Classics in abdominal radiology: the jumping deer sign

**DOI:** 10.1007/s00261-024-04699-6

**Published:** 2024-11-22

**Authors:** Samantha Elliott, Trent Taros, Adam Lustig

**Affiliations:** Eastern Virginia Medical School, Norfolk, USA

**Keywords:** Classics in abdominal radiology, Hepatobiliary, Jumping deer, Jumping stag, Liver, Normal anatomy, Ultrasound, Radiology signs, Signs

## Abstract

The “jumping deer sign” is an ultrasonographic pattern that aids in identifying normal liver anatomy and distinguishing it from pathology. It includes the portal vein (deer’s head and body), the gallbladder or cystic duct (tail), and the inferior vena cava (obstacle). This sign helps differentiate portal veins from intrahepatic ducts, crucial for diagnosing conditions like portal hypertension. It also assists in identifying gallbladder pathologies and assessing the IVC for hydration status. The jumping deer sign provides a clear reference for clinicians, improving diagnostic accuracy, especially for those with limited ultrasound experience.

The “jumping deer sign” is a finding on liver ultrasonography representing normal anatomic relationships of often confusing tubular structures within the liver parenchyma. The structures that make up and are identified as parts of the jumping deer sign include the portal vein and its primary branches, the gallbladder (or cystic duct leading to the gallbladder), and the IVC (Fig. 1). Signs and other memory or pattern recognition heuristics are essential to identifying and characterizing pathology. Just as important, but much rarer, are those reassuring signs, whose presence is reassuring rather than frightening. The “jumping deer sign” is one such sign when evaluating for liver conditions that may distort adjacent structures.

The portal vein and its primary branches comprise the head and body of the jumping deer (Fig. 2a). The anatomy of the portal venous system can vary widely, which may lead to misinterpretations and errors when distinguishing portal veins from intrahepatic ducts or systemic veins. Accurately differentiating portal veins from bile ducts is crucial for evaluating conditions such as portal hypertension, primary biliary cholangitis, and primary sclerosing cholangitis. While portal hypertension is typically imaged extrahepatically on ultrasound, it can also be identified intrahepatically by the presence of a dilated portal vein or hepatofugal (reversed) blood flow. Clinical signs such as ascites, varices, and splenomegaly further support the diagnosis. Conversely, liver pathologies involving the intrahepatic biliary ducts may present with distinct sonographic features, including intrahepatic ductal dilatation and thickened ductal walls [1]. Therefore, understanding normal portal venous anatomy is crucial for accurately identifying these pathologies. Doppler ultrasound can be a valuable tool for differentiating portal veins from bile ducts, but a solid grasp of the underlying anatomy remains essential. Finding the jumping deer sign on ultrasound can help confirm the veracity of presumed anatomical structures and reduce the risk of confusing portal veins with dilated ducts.

**Fig. 1:**
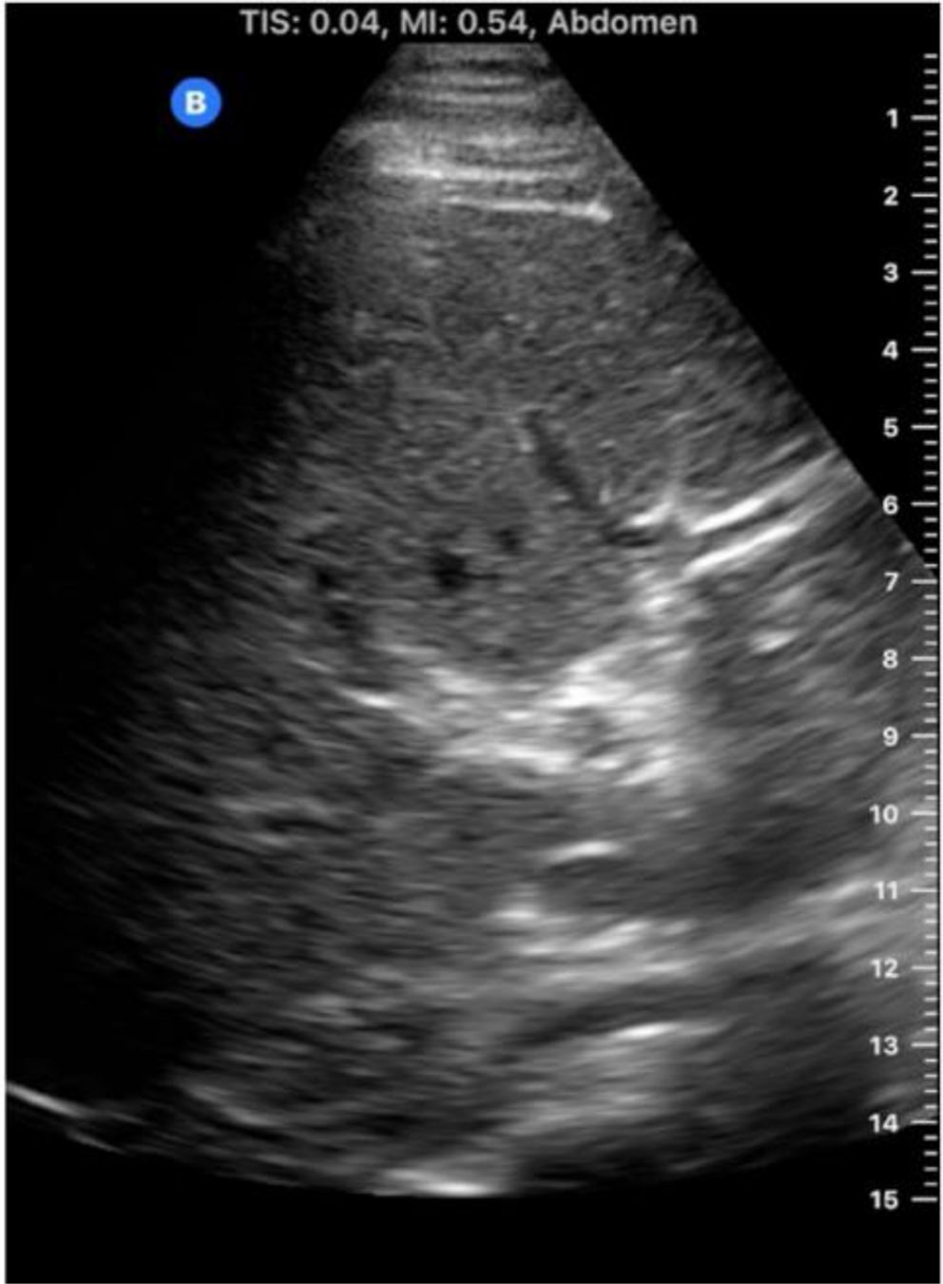
M mode transverse ultrasound of the liver depicting the “jumping deer sign”

As the “deer” leaps forward over the “log”, it is trailed by its bushy, somewhat bulbous tail, a structure that is the gallbladder (or cystic duct) (Fig. 2a). Ultrasound is a highly effective imaging modality for detecting gallbladder pathologies. Acute cholecystitis and biliary colic are among the most common reasons for emergency department visits, with right upper quadrant ultrasound being the diagnostic tool of choice due to its accessibility, non-invasiveness and ability to provide detailed images of the gallbladder and surrounding structures [2]. In addition, emphysematous cholecystitis, an often life-threatening condition with a mortality rate of up to 25%, is caused by gas-forming bacteria that infects the gallbladder wall [3]. This condition may present as echogenic foci with posterior dirty shadowing or reverberation artifacts within the gallbladder wall. Unfortunately, this is very similar in appearance to bowel gas, with posterior shadowing obscuring detail behind said gas and potentially leading to confusing one for the other. Identifying the normal anatomy, made easier by seeing the jumping deer’s tail, may help differentiate emphysematous cholecystitis from bowel gas. Furthermore, a “clipped tail” in the ultrasound image may suggest a cholecystectomy, particularly if surgical clips are visible in the area where the tail would normally be.

**Fig. 2:**
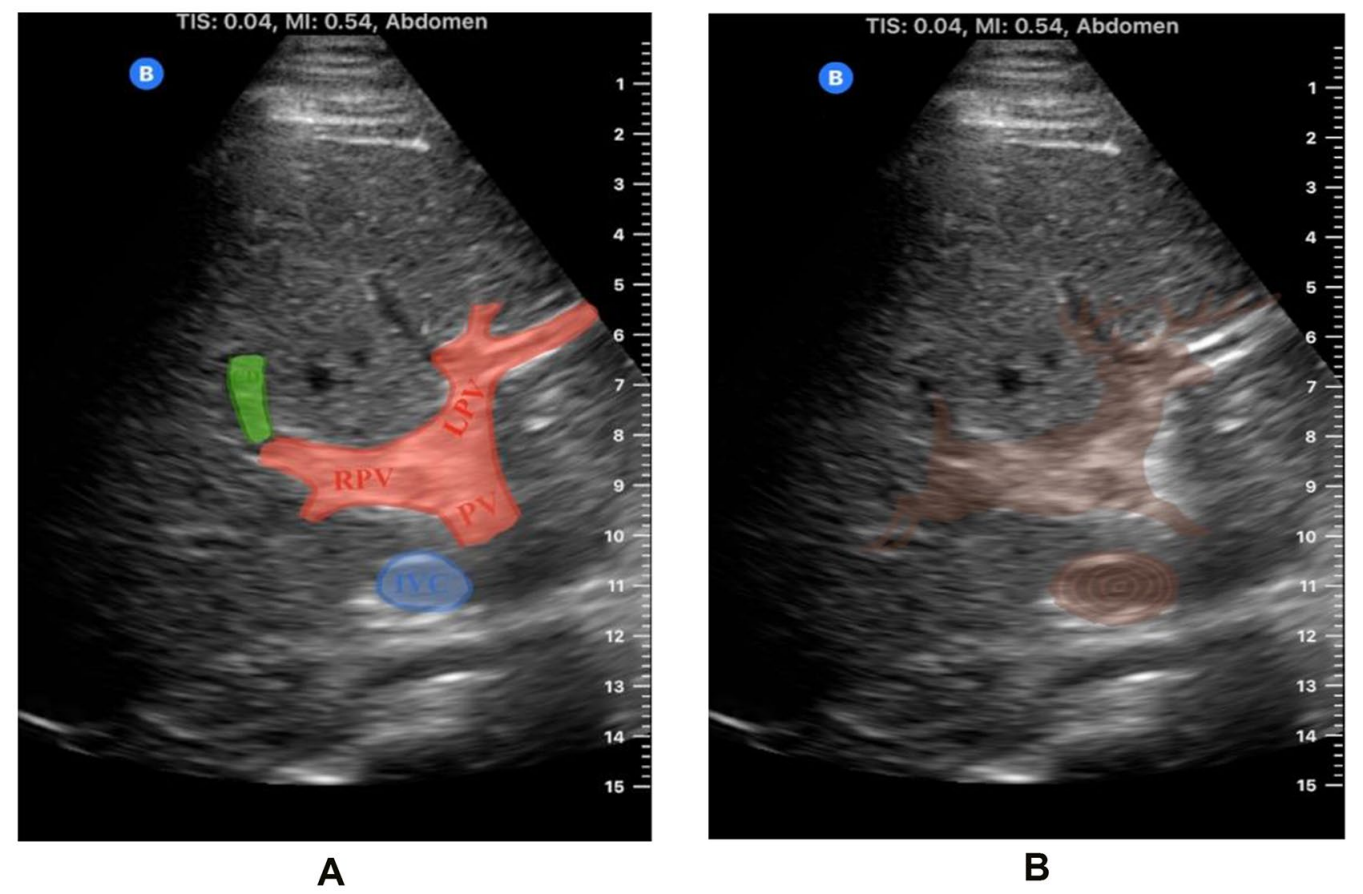
(**A**) Ultrasound image demonstrating the “jumping deer sign” with overlays to accentuate anatomical structures: red overlay highlighting the body and head of the deer represented by the main portal vein (PV), right portal vein (RPV) and left portal vein (LPV), green overlay representing the cystic duct (CD), and blue overlay delineating the inferior vena cava (IVC). (**B**) Abdominal ultrasound image featuring the “jumping deer sign” with a brown overlay illustrating the deer (portal vein and cystic duct leading to the gallbladder) leaping over the log (IVC)

Finally, the obstacle over which the “deer” can be seen jumping is the inferior vena cava (IVC) (Fig. 2b). Measurement of the IVC using ultrasound is a valuable tool for assessing hydration status, especially in critically ill patients. The size and collapsibility of the IVC can provide insights into intravascular volume and right atrial pressure [4]. A distended, non-collapsible IVC may indicate fluid overload or elevated central venous pressure (CVP), which is often seen in conditions such as heart failure. Conversely, a small, collapsible IVC usually suggests low intravascular volume and hypovolemia. Therefore, assessing IVC collapsibility is crucial for management decisions. A recent study found that sonographic IVC diameter measurements are accurate and correlate well with direct CVP measurements made by catheterization, which had previously been the gold standard [5]. These results suggest that sonographic IVC measurement could potentially replace more invasive techniques. Easy and rapid identification of the IVC in a window not obscured by bowel gas, such as that offered intrahepatically via the jumping deer sign, is therefore very advantageous in the management of acutely ill patients.

In conclusion, the “jumping deer sign” offers a unique and valuable framework for interpreting liver ultrasonography, enhancing diagnostic accuracy, and reducing potential misinterpretations of liver anatomy. The jumping deer sign is particularly beneficial for clinicians with less ultrasound experience, as it provides a clear and recognizable anatomical reference. By recognizing the portal vein, gallbladder (or cystic duct), and inferior vena cava, clinicians can better distinguish between normal anatomical variations and pathologic conditions. Overall, the “jumping deer sign” demonstrates the utility of pattern recognition in ultrasound, offering both clarity and reassurance in the complex field of liver imaging.

## Data Availability

No datasets were generated or analysed during the current study.
